# Predicting Kinase Activity in Angiotensin Receptor Phosphoproteomes Based on Sequence-Motifs and Interactions

**DOI:** 10.1371/journal.pone.0094672

**Published:** 2014-04-10

**Authors:** Rikke Bøgebo, Heiko Horn, Jesper V. Olsen, Steen Gammeltoft, Lars J. Jensen, Jakob L. Hansen, Gitte L. Christensen

**Affiliations:** 1 Department of Clinical Biochemistry, Glostrup Research Institute, Glostrup Hospital, Glostrup, Denmark; 2 Department of Disease Systems Biology, Novo Nordisk Foundation Center for Protein Research, University of Copenhagen, Copenhagen, Denmark; 3 Department of Proteomics, Novo Nordisk Foundation Center for Protein Research, University of Copenhagen, Copenhagen, Denmark; 4 Department of Biomedical Sciences and The Danish National Research Foundation Centre for Cardiac Arrhythmia, Faculty of Health Sciences, University of Copenhagen, Copenhagen, Denmark; 5 Cellular and Metabolic Research Section, Department of Biomedical Sciences, University of Copenhagen, Copenhagen, Denmark; University of São Paulo, Brazil

## Abstract

Recent progress in the understanding of seven-transmembrane receptor (7TMR) signalling has promoted the development of a new generation of pathway selective ligands. The angiotensin II type I receptor (AT_1a_R) is one of the most studied 7TMRs with respect to selective activation of the β-arrestin dependent signalling. Two complimentary global phosphoproteomics studies have analyzed the complex signalling induced by the AT_1a_R. Here we integrate the data sets from these studies and perform a joint analysis using a novel method for prediction of differential kinase activity from phosphoproteomics data. The method builds upon NetworKIN, which applies sophisticated linear motif analysis in combination with contextual network modelling to predict kinase-substrate associations with high accuracy and sensitivity. These predictions form the basis for subsequently nonparametric statistical analysis to identify likely activated kinases. This suggested that AT_1a_R-dependent signalling activates 48 of the 285 kinases detected in HEK293 cells. Of these, Aurora B, CLK3 and PKG1 have not previously been described in the pathway whereas others, such as PKA, PKB and PKC, are well known. In summary, we have developed a new method for kinase-centric analysis of phosphoproteomes to pinpoint differential kinase activity in large-scale data sets.

## Introduction

Getting an overview of the complex propagation of cellular signal transduction is important to understand the process from receptor activation to phenotypic outcomes. Protein phosphorylation is central to cellular signalling and can be systematically investigated using quantitative mass spectrometry (MS) [1], [2]. Global analysis of ligand induced changes in phosphorylation can be achieved using stable isotopic labelling of amino acids in cell culture (SILAC)[2]. In a typical SILAC setup, two or three cell cultures are grown in parallel, one on a medium with regular amino acids and one or two on a medium with isotopically labelled amino acids. Once the cell cultures have almost fully incorporated the isotopic labelled amino into their proteomes, they can be differentially stimulated, lysed, mixed and jointly analysed in the mass spectrometer to minimize unwanted biases. Peptides from the two experimental conditions can be differentiated from the known molecular weight difference arising from the labelled amino acids [3]. In this study, we present a joint analysis of two complimentary SILAC-based phosphoproteomics studies that have portrayed the complex signalling induced by the angiotensin II type 1 receptor (AT_1a_R) [4], [5].

**Figure 1:**
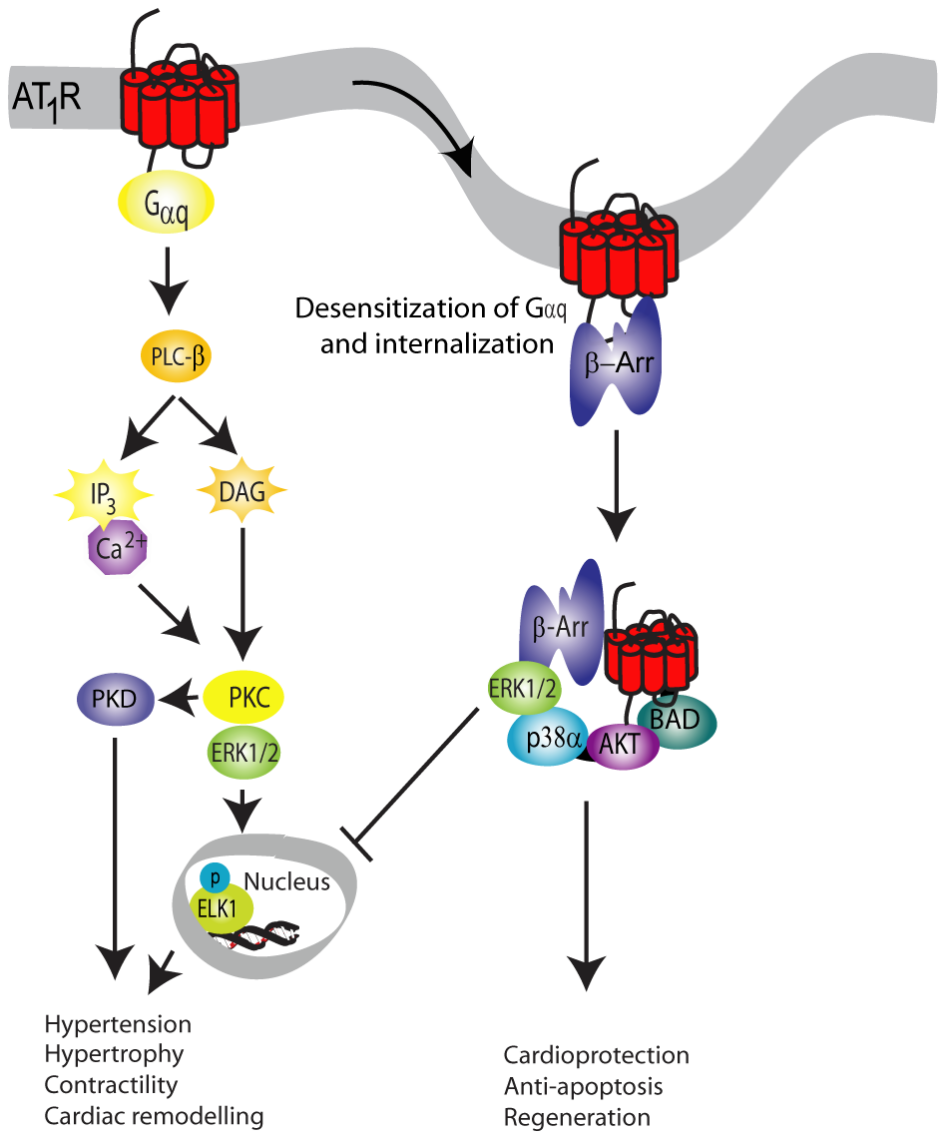
The angiotensin II signalling pathway. The angiotensin II signalling pathway can signal through G-protein dependent and G-protein independent pathways. The angiotensin receptor (AT_1a_R) is phosphorylated upon stimulation and G-proteins activated, which leads to an increase in IP3, intracellular calcium and diacyglycerol, which activates the signalling cascade involving several kinases e.g. PKC, MAPK and PKD. Phosphorylation of the receptor also leads to the recruitment of G-protein coupled receptor kinases and β-arrestin, and ultimately to internalisation of the receptor. Internalisation of the receptor yields the second wave of signalling, and has been associated with some of the positive effects of angiotensin II signalling in the cardiac setting.

AT_1a_R (Figure 1) is an important cardiovascular seven transmembrane receptor (7TMR). It has been one of the first and most important receptors for defining the concept of functional selectivity, i.e. that selective ligands can have agonistic effects on one signalling pathway while antagonizing another [7]. Traditionally, drugs that target 7TMRs have been described as either agonists or antagonists, based on their ability to induce or inhibit G-protein dependent signalling. The discovery that one ligand can differentially affect multiple signalling pathways represents an enormous potential for the development of drugs which might have less side effects or be more efficacious. Biased agonists inhibiting the AT_1a_R G-protein dependent signalling while preserving β-arrestin signalling have a promising profile for treatment of cardiac diseases as they largely separate the G-protein initiated hypertensive and hypertrophic effects from the β-arrestin-mediated cardioprotective and regenerative mechanisms [8]–[10]. Although much is known about the molecular mechanisms leading to functional ligand selectivity and the first steps in the separation of the major signalling pathways, it remains challenging to get an overview of the complex signalling induced by the AT_1a_R. Christensen and co-workers compared the effect of the natural agonist Ang II and the β-arrestin selective agonist SII Ang II [4], whereas Xiao and co-workers focused on the signalling initiated by SII Ang II [5]. The two studies were designed similar and are thus comparable. Due to the partly stochastic nature of mass spectrometry [6] and minor differences in peptide isolation methods used in the studies, combining the two studies can provide a more comprehensive description of the AT_1a_R signalling than any of the studies alone.

To gain further insight into the function of the modifications identified in phosphoproteomics studies, multiple approaches can be used to predict the activity of kinases based on the regulated phosphopeptides. The activities of many kinases are themselves regulated through phosphorylation, and Xiao and co-workers supplemented their mass spectrometry data with an antibody array against known regulatory phosphorylation sites on a panel of kinases; however, both datasets contain many phosphorylation sites on kinases, for which the effect on kinase activity remains to be elucidated. Algorithms like the kinase enrichment analysis compare the regulated proteins to a background database of known interactions [11]. As this approach relies solely on protein interaction and not specific phosphosites, it disregards the fact that the majority of substrates can have several phosphosites and that these sites are not necessarily targeted by the same kinase. Conversely, tools like Motif-X [12], which identify over-represented sequence motifs, are able to differentiate between multiple modification sites but ignore the cellular context [13]–[15]. However, it is difficult to pinpoint the specific kinases, as many of them recognise substrates with similar or identical sequence motifs [16].

To address these issues, the NetworKIN algorithm combines sequence motifs with data on cellular context in the form of protein interactions to reliably assign kinases to their corresponding modification sites [17]. Here, we present an approach that compares the NetworKIN score distributions for each kinase under two conditions to identify kinases with differential activity. We illustrate the power of this approach by applying it to the combined phosphoproteomics data set on AT_1a_R -mediated signalling.

## Results and Discussion

### Combining the two AT_1a_R phosphoproteomes

The phosphoproteomics study of AT_1a_R signalling by Christensen and co-workers reported 1182 individual phosphosites on 530 proteins to be up-regulated by Ang II or SII Ang II [4]. Hereof, the SII Ang II dependent subset; induced by the β-arrestin biased agonist SII Ang II is 36% or 424 phosphosites on 204 proteins. The study by Xiao and co-workers, which focused on β-arrestin dependent signalling induced by SII Ang II stimulation, revealed 207 up-regulated phosphosites on 146 unique proteins[5]. The two studies use different methods to enrich for phosphopeptides, namely immobilized metal affinity chromatography (IMAC) and titanium dioxide (TiO_2_). Although both methods generate reproducible results, the concentrations used vary slightly as do the stimulation time. In addition, they have different preferences for phosphopeptides, and are thus complementary to each other [18].

**Figure 2:**
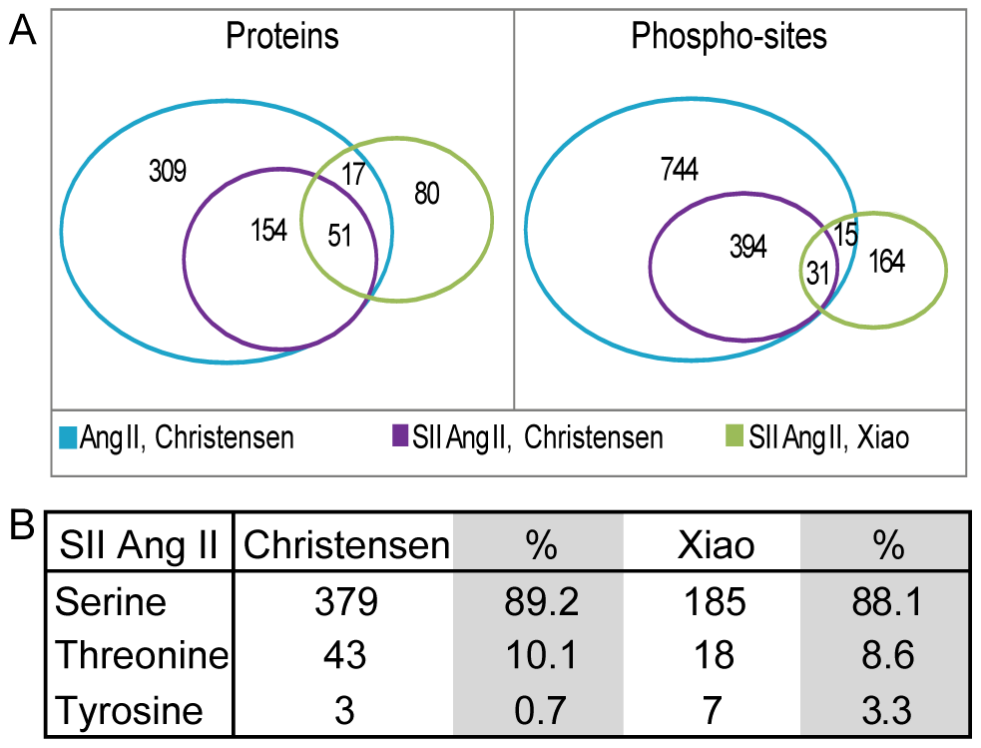
Comparison of the up-regulated proteins and phosphosites detected in the two MS studies. The IPI IDs of the dataset from Xiao and co-workers was mapped to UniProt, and the two ID lists were compared by an in-house script. A) Comparison of the two SII Ang II regulated data sets revealed surprisingly little overlap in phosphosites, only 5%. On the protein level the overlap was merely 17%. The figure also shows that 15 phosphosites detected to be regulated only with Ang II in the Christensen study was regulated with SII Ang II in the study by Xiao and co-workers. Of these 15 phosphosites, 5 were more than 1.4 fold regulated, and was included as SII Ang II regulated in the analysis. B) The distribution of Ser, Tyr and Thr sites in the two studies.

**Figure 3:**
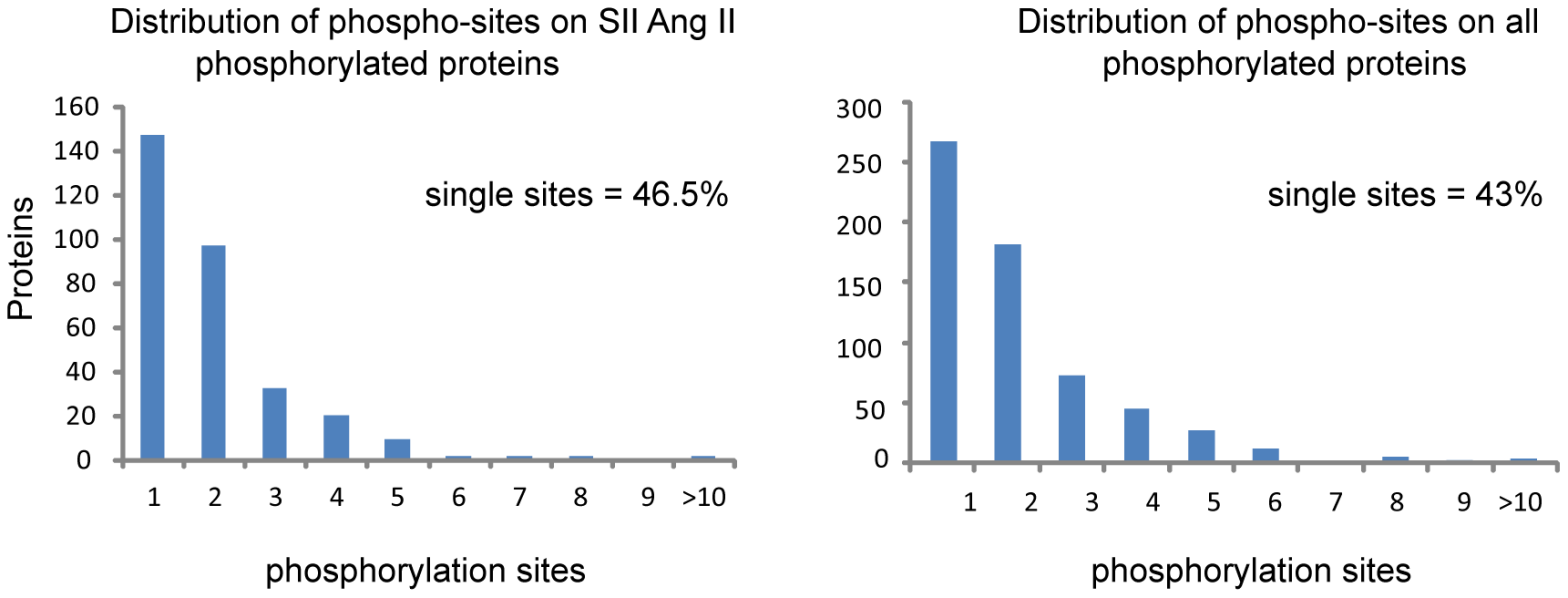
Distribution of phosphosites. The percentage of proteins with multiple phosphosites in the angiotensin phosphoproteome is 57% and the number of proteins with multiple phosphosites decreases with increasing number of regulated phosphosites. The same distribution is observed when analysing the SII Ang II regulated subset.

We combined both studies to obtain a total of 1339 up-regulated phosphosites on 605 proteins. The SII Ang II dependent signalling subset comprised of 600 SII Ang II up-regulated phosphosites on 299 proteins (Table S1). As expected given the biological and experimental differences between the two studies, the overlap in SII Ang II dependent signalling is small: only 31 of the 600 phosphosites (5%) were found in both studies. However, the overlap is considerably higher at the level of proteins, with 51 of the 299 phosphorylated proteins (17%) being identified in both studies (Figure 2A). This shows that many proteins involved in β-arrestin dependent AT_1a_R signalling are phosphorylated on several residues, with the two studies identifying different sites in the same proteins. On average two phosphosites were identified per protein; the distribution of the number of phosphosites per protein is shown in Figure 3.

### Prediction of differences in kinase activity profiles

**Figure 4:**
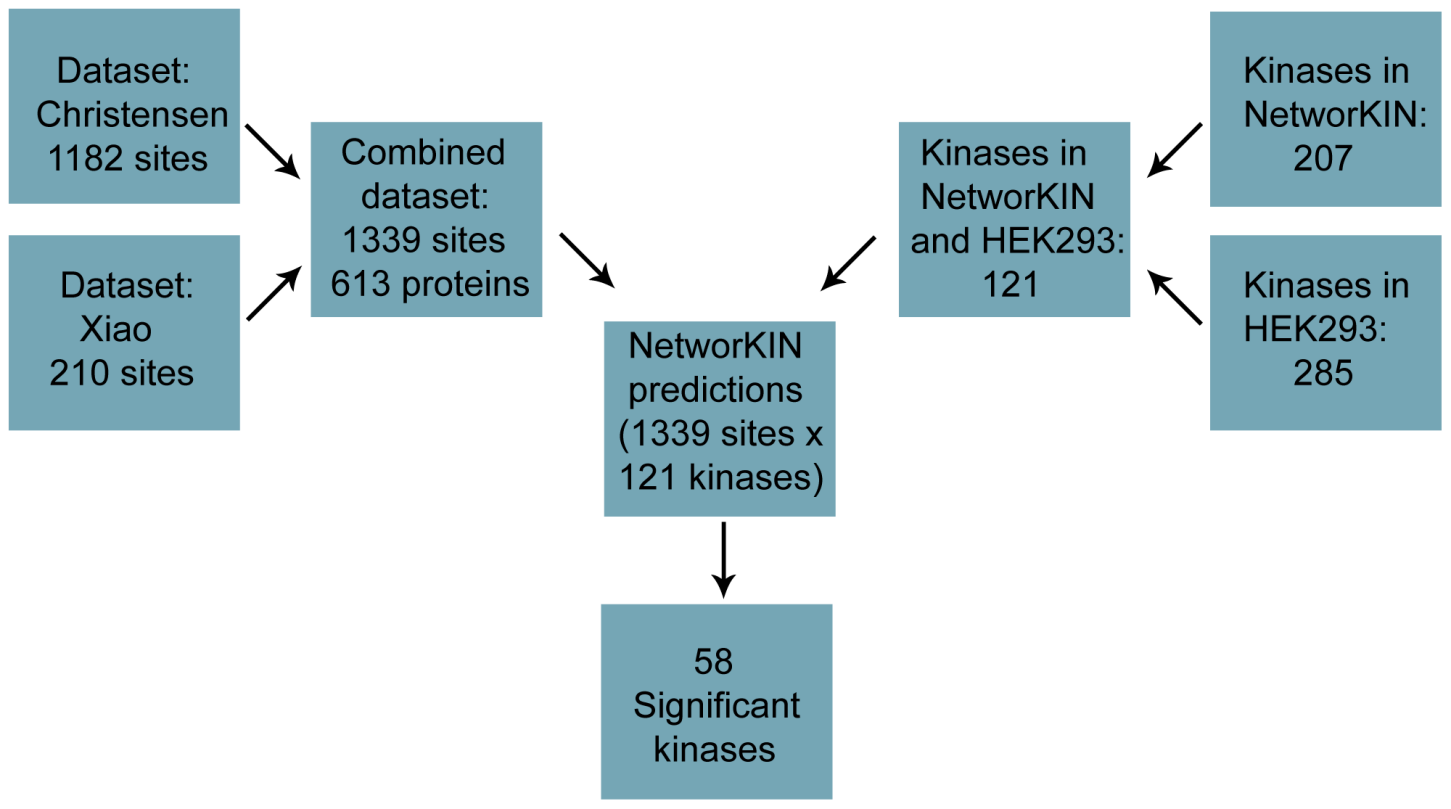
The flow of phosphosites and kinases. Data was collected from Xiao et al., 2010 and Christensen et al., 2010. The combination of the two data sets resulted in a list of 1339 up-regulated phosphosites, which were used for prediction of activated kinases based on the NetworKIN algorithm. The 207 kinases in NetworKIN were reduced to 59, which were detected by mass spectrometry in the AT_1a_R-HEK293 proteome, had enough predictions for statistical analysis and a statistically significant p-value.

To predict the differential activity of kinases, we run the NetworKIN algorithm on all 1339 up-regulated sites identified in the studies (Table S2). NetworKIN covers a list of 207 protein kinases, however not all may be present in HEK293 cells. In a separate experiment we examined the expression of kinases in AT_1a_R HEK293 cells (Table S3). By combining this experiment and the two proteomes provided by Xiao and Christensen, we found a total of 285 protein kinases to be expressed in AT_1a_R HEK293 cells. Comparing the list of the detected protein kinases to the kinases included in NetworKIN (Table S3) showed that 121 of the 285 detected protein kinases are included in NetworKIN. To account for this, we filtered the prediction results to include only protein kinases known to be expressed in the HEK293 cells in the analysis. Figure 4 illustrates the overlap of the kinases found in the studies with the kinases supported by the NetworKIN algorithm.

**Figure 5:**
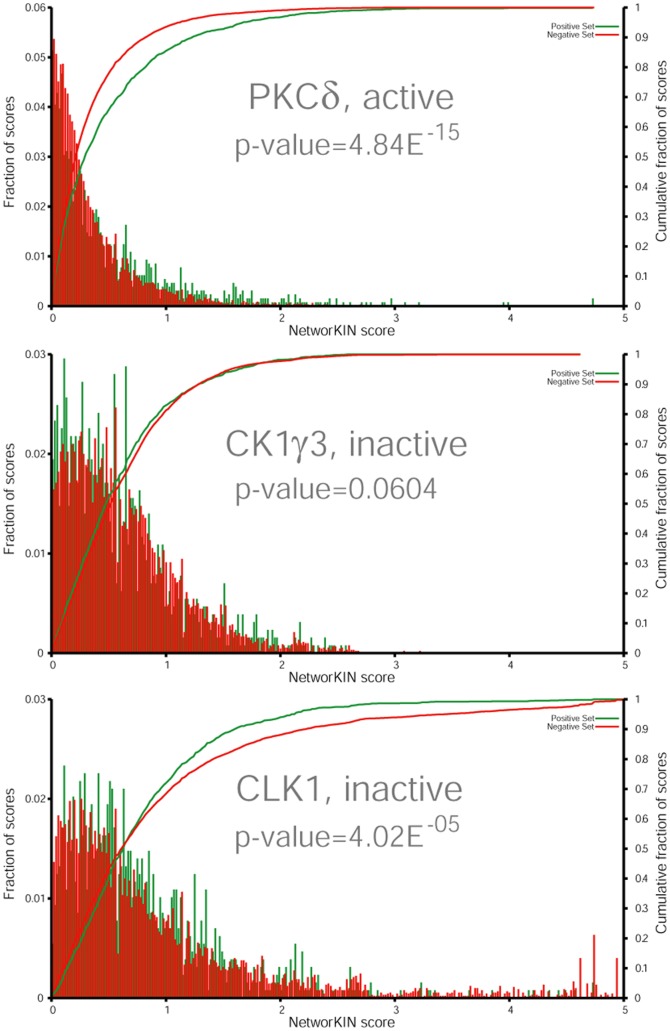
Score distribution plots for the regulated versus non-regulated phosphosites of three kinases. The score for each phosphosite was predicted by NetworKIN, both for regulated and non-regulated phosphosites. Due to the difference in sites in the two groups (10x), the counts were normalized to percentage. The likelihood of having a phosphosite of a given score is indicated by a bar, green indicates regulated phosphosites and red the non-regulated sites. In the top plot the score distributions are significantly different and in favour of the regulated phosphosites. Thus PKCδ is an active kinase. CK1γ3 resembles an inactive kinase, the score distributions are similarly and the p-value is non-significant. The bottom plot have a significantly different score distribution, however this is in favour of the non-regulated sites, and therefore an inactive kinase.

NetworKIN scores represent how likely a site is to be modified by a specific kinase. By comparing the NetworKIN score distributions for the same kinase under two different conditions, we can identify statistically significant differences and thereby predict changes in kinase activity between the conditions (Figure S1). The scores in NetworKIN are relative scores, normalized for the proteome-wide score distribution for each kinase specific predictor. Scores are furthermore log-transformed to increase the resolution in the high scoring range: a score of 2 or 3 corresponds to a prediction better than 99.9% or 99.99%, respectively, of all scores in the proteome for the specific kinase. The plot for PKCδ (Figure 5a) exemplifies a kinase predicted to be active, which is reflected in a significant shift towards higher scores for the up-regulated sites. By contrast, the score distributions for CK1γ3 (Figure 5b) illustrate a non-regulated kinase, with a non significant difference between the regulated and non-regulated sites. The analysis also allows for detection of down-regulated kinases like CLK1 (Figure 5c), which shows a shift towards lower scores for the regulated sites.

**Figure 6:**
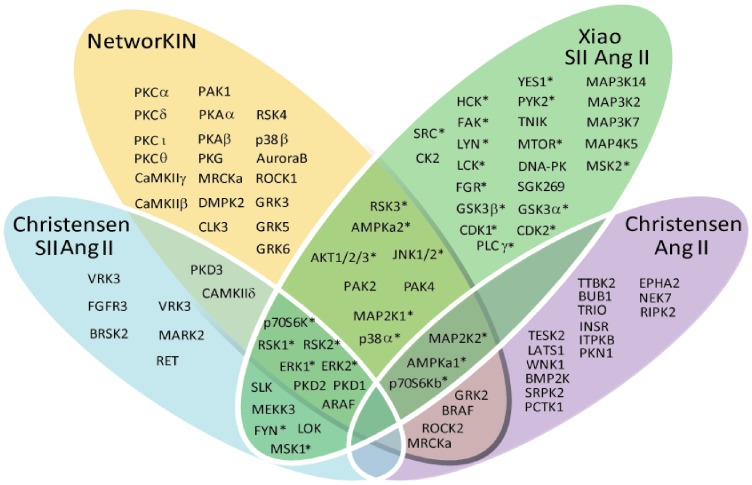
Kinases involved in the angiotensin signalling pathway. A comparison of the kinases phosphorylated in Christensen et al., 2010 and Xiao et al., 2010, with an overlay of the NetworKIN predicted active kinases. More than 30 of the kinases were differentially regulated on a phosphosite involved in kinase activity (indicated with *).

The statistical analysis of up-regulated versus non-regulated phosphosites suggested increased activity for 48 and decreased activity for 10 of the 121 kinases (Table 1, Figure 6). The vast majority (89%) of the 48 activated kinases are known to be involved in angiotensin II signalling, validating the approach, and 26 of them harbour regulated phosphosites reported in the two studies (P<0.0001; Fishers exact test). Among these we found ERK1/2, RSK2 and p70S6, for which the phosphosites have been linked to kinase activity. Furthermore, the method suggested increased activity of well-known Ang II regulated kinases, e.g. GRK5/6 and PKC, which were not found to be phosphorylated themselves.

**Table 1:** NetworKIN predicted active kinases.

Name	Phosphosites	Ref
AKT1	Ser129, Thr308*	5
AKT2		
AKT3		
AMPKa1	Ser497;Thr183*	4; 5
AMPKa2	Thr172*	5
ARAF	Thr181, Ser186	4
**AuroraB**		
BRAF	Ser151	4
CaMKIIβ		
CaMKIIδ	Ser330, Ser333, Ser334, Ser337	4
CaMKIIγ		
**CLK3**		
**DMPK2**		
MRCKa	Ser1673, Ser1676	4
**MRCKb**		
GRK2	Ser685	4
GRK3		
GRK5		
GRK6		
JNK1	Thr183*,Tyr185*	5
JNK2	Thr183*,Tyr185*	5
MAP2K1	Ser218*,Ser222*	5
MAP2K2	Thr394, Thr396 Ser222*,Ser226*	4;5
ERK1	Thr198, Thr202*, Tyr204*	4
ERK2	Thr185*, Tyr187*	4
p38α	Thr180*, Tyr182*	5
p38β		
p70S6K	Ser441, Thr444, Ser205; Thr252*, Ser394*, Thr412*	4
p70S6Kb	Ser416, Ser417, Ser423; Thr228*, Ser370*, Thr388*	4
PAK1		
PAK2	Thr169	5
PAK4	Ser291	5
PKCα		
PKCδ		
PKCι		
PKCθ		
PKD1	Ser205, Ser208, Ser210, Ser237, Thr395, Ser397, Ser401; S742*	4; 5
PKD2	Ser197, Ser198, Thr199, Ser203, Ser396	4
PKD3	Ser41, Ser44	4
**PKG1**		
PKAα		
PKAβ		
ROCK1		
ROCK2	Ser1137	4
RSK1	Thr359, Ser363, Ser369, Ser372, Ser733; Ser221*, Ser380*	4; 5
RSK2	Thr365, Ser369, Ser375; S227*, S386*	4; 5
RSK3	Ser377*	5
RSK4		

Phosphorylation sites from the two proteomic studies were analysed using the NetworKIN algorithm and the predictions were filtered based on kinases detected in the AT_1a_R-HEK kinome. NetworKIN verified the activity of many of the kinases found by phospho-antibody array by Xiao et al., 2010 and predicted activity for several other kinases, of which around half have been described in the literature of angiotensin II signalling. Numbering of amino acids is according to SwissProt. Amino acids marked * are involved in the regulation of the kinase (SwissProt). Kinases that have not previously been described to be regulated in the angiotensin II signaling pathway are depicted in bold. Phosphorylation sites detected in either of the two proteomic studies Christensen et al., 2010 and Xiao et al., 2010 are denoted Ref. 4 and 5, respectively.

Three of the kinases we predict to be activated had not previously been described as regulated by Ang II or as involved in cardiac pathology when the analysis was performed. These are Aurora kinase B (Aurora B), Dual specificity protein kinase CLK3 and cGMP-dependent protein kinase 1 (PKG1). Recently, Aurora B was shown to phosphorylate certain histone deacetylases (HDACs) with critical roles in cardiac disease and thereby likely regulate their localization [19]. PKG1 is involved in vascular smooth muscle relaxation, where it influences actin binding by the phosphorylation of proteins important for cytoskeletal reorganisation [20] which fits well with regulation by Ang II. In addition to predicting active kinases, the statistical analysis of the NetworKIN predictions revealed that the p-value for some kinases were non-significant, indicating that their activity were neither up- nor down-regulated despite Ang II induced phosphorylation of the kinase (e.g. GSK3α, GSK3β, and CDK1).

Despite the lack of up-regulated phosphosites on the constitutively active kinase CK2 our statistical analysis suggests that CK2a and CK2a2 are both inactivated (Figure S1 and Table S4). The statistical analysis furthermore predicts reduced activity of the kinases ATM, ATR, CDK2, CDK3, CK2, CLK1, CLK4 and TLK1.

### Tyrosine phosphorylation in the AT_1a_R signalosome

Several earlier studies on AT_1a_R signalling have described angiotensin II mediated tyrosine phosphorylation [21]–[25]. Proteins harbouring these are often low abundant [2] and in addition current enrichment methods favour peptides phosphorylated on serines or threonines [18]. This leads to tyrosine phosphorylation being significantly underreported; in the two studies the fraction of tyrosine sites was 0.7% and 3.3% for the studies by Christensen and co-workers and Xiao and co-workers respectively (Figure 2B). However, by analysing a human phosphorylation-antibody array, Xiao and co-workers showed the activation of several tyrosine kinases in response to SII Ang II stimulation (SRC, FAK, HCK, FGR, LYN, LCK, and YES1), indicating that the bias against tyrosine sites indeed arises from experimental limitations.

### Analysis of the phosphoproteome reveals multiple regulated parallel pathways

To generate an overview of the AT_1a_R signalling in the form of pathway maps, we performed enrichment analysis of all 605 proteins. The enrichment analysis searches for a larger-than-expected overlap between the input list of proteins and e.g. the members of a known pathway. If a statistically significant number of proteins from the query list are members of the pathway, it may indicate that this pathway plays a role in the studied condition. We analysed the combined data set using the MetaCore algorithm (http://www.genego.com/metacore.php). However, the algorithm was only able to map 265 of the 605 proteins, indicating that several proteins remain to be assigned a function in a signalling context in the MetaCore algorithm.

The analysis revealed that a number of signalling pathways are regulated by angiotensin II. Among these were pathways previously implicated in cross-talk with angiotensin II signalling, namely insulin [26] and EGFR [27], [28] signalling. The data also points to angiotensin II mediated regulation of the WNT pathway [29] (AKT, APC, GSK3β, β-catenin, TCF7L2 and Bcl-9-2). The 10 highest-ranking pathways are shown in Table 2. The most significant enriched pathway was the G-protein coupled receptor Gastrin. AT_1a_R and the Gastrin receptor both signal through the same G-proteins (G_αq/11_) and share many proteins in their downstream signalling cascades. This explains the presence of many additional GPCR signalling pathways in the list of the significantly enriched pathways. However, angiotensin II signalling was not among the list of these, suggesting that according to MetaCore, a minority of the phosphorylated proteins are described in the pathway. In MetaCore the angiotensin II pathway have been divided into 2 maps, the “Angiotensin signalling via β-arrestin” and “Angiotensin via PYK” (the 2 maps are available at http://pathwaymaps.com/maps/, however not all maps in the MetaCore algorithm are freely available). We merged the two maps, thereby creating one map for the angiotensin II pathway, thus illustrating how many of the involved proteins were regulated. Reanalysis of the enrichment analysis including the merged angiotensin II pathway map, placed the angiotensin II pathway map as the top-scoring map.

**Table 2:** The AT1aR regulated pathway maps.

Group	Name of the map	pValue	ratio
Development	Gastrin in cell growth and proliferation	5,09E-18	22/62
Cytoskeleton remodeling	Cytoskeleton remodeling	5,91E-15	24/102
Development	FGFR signaling pathway	1,46E-14	18/53
Cytoskeleton remodeling	TGF, WNT and cytoskeletal remodeling	4,51E-14	24/111
Development	Flt3 signaling	1,29E-13	16/44
Development	Thrombopoietin-regulated cell processes	1,94E-13	16/45
Regulation of lipid metabolism	Insulin signaling:generic cascades	4,26E-13	16/47
Chemotaxis	CXCR4 signaling pathway	5,98E-13	14/34
Development	A2B receptor: action via Gprotein alpha s	1,28E-12	16/50
Immune response	IL15 signaling	7,41E-12	17/64

The phosphopeptides regulated by Ang II or/and SII Ang II was used for enrichment analysis in MetaCore (GeneGo). The enrichment analysis retrieved a list of pathways from the database. The 10 most significant pathways are shown. The majority of the pathways are in good agreement with the current knowledge of angiotensin II signalling.

To illustrate the interconnection and the broad overlap between the top-5 scoring pathways and the merged angiotensin II pathway map, we combined the maps in MetaCore. In order to aid the clarity of the combined map, unregulated parts of top-5 scoring pathways were removed. This illustrates an overview of the regulated proteins and their interconnections (Figure S2).The integrated map shows, that 19 of the 43 proteins on the angiotensin II pathway map are regulated by both G-protein dependent and independent signalling (Figure S2). This is in agreement with previous studies, which have shown that both signalling mechanisms activate the MAP kinases ERK1/2 [30]. Despite what we know of separate G-protein and β-arrestin dependent pathways initiated by AT_1a_R [30]-, the map does not contain sub clusters of proteins solely regulated by the G-protein dependent Ang II pathway.

Previous studies have shown that the MAP kinases ERK1/2 are activated by both G-protein dependent and independent signalling mechanisms [30]. G-protein dependent Ang II signalling leads to nuclear localisation of ERK1/2, whereas ERK1/2 activated by G-protein independent SII Ang II stimulus is restricted to the cytoplasm, an example of two parallel pathways affecting the same signalling proteins. G-protein independent signal transduction has been linked to cell survival and renewal [33], [34], but do surprisingly not regulate significant gene expression [35], [36]. The exact signalling pathway giving rise to G-protein independent nuclear effects mediating proliferative effects remain to be elucidated. In summary, we present a novel workflow for the prediction of differential active kinases by statistically comparing the NetworKIN scores in the non-stimulated and stimulated conditions. The NetworKIN usage of phosphosites as “fingerprints” for activity of kinases in combination with knowledge on protein-protein interaction provides additional valuable information about the signalling backbone in the original phosphoproteomics analysis. The confidence in our results is strengthened by the fact, that we predict kinases well known to be regulated by angiotensin II. Overall, this study provides a global unbiased overview of the AT_1a_R signaling, hereunder the G-protein dependent and – independent components, and predicts a new set of kinases and other target proteins to study in the coming years, which will be helpful in directing research to increase the knowledge of this important cardiovascular regulator.

## Materials and Methods

### Data description

We mapped the list of regulated phosphosites from Xiao and co-workers [5] to UniProt sequences and subsequently combined them with the results of Christensen and co-workers [4]. Xiao and co-workers [5] used the Ambiguity score algorithm for localization of phosphosites, while Christensen and colleagues [4] used the Mascot decoy score. Since the scope of this paper is not to evaluate these two algorithms, we have chosen to rely on the authors' confidence in the used algorithms and thus localization of the phosphosites.

In the case of contradictory results for the regulation of phosphorylation sites, e.g. sites reported as up-regulated in one and detected but not up-regulated in the other study, we curated the data. We defined these borderline significant sites as regulated, if the fold-change in the study reporting it as not up-regulated was higher than 1.4.

### NetworKIN

The 1339 up-regulated phosphosites were analysed by NetworKIN v3 [17]. The NetworKIN algorithm predicts kinase-substrate interactions based on sequence motifs and cellular context. The NetworKIN results are relative scores, normalized for the proteome-wide score distribution for each kinase-specific predictor. Scores are furthermore log-transformed to increase the resolution in the high scoring range. A score of 2 or 3 corresponds to a prediction better than 99.9% or 99.99% respectively of all scores in the proteome for the specific kinase.

### Statistics

As background data set we used the prediction of the 9500 non-regulated phosphosites published in [4]. The Kolmogorov-Smirnov test (KS test) was used to compare score distributions between the two sets. Kolmogorov-Smirnov is a nonparametric test for the equality of continuous one-dimensional probability distributions. The p-value indicates how alike the background distribution and the up-regulated score distribution for a given kinase is. A smaller p-value indicates a more significant difference in the distributions. With a p-value less than 0.05 we expect that in 5 of 100 instances we would wrongly claim two equal distributions to be different, thus wrongly claiming the kinase differential active. As distributions for 121 kinases were analysed, it is likely that by random chance we find kinases with significant difference in distributions. In order to reduce this we correct for multiple testing by correcting the p-values for false discovery rate correction (Benjamini-Hochberg method). A stringent p-value of 0.005 was applied. To discriminate between active and inactive kinases, we calculated p-values for the likelihood of the regulated phosphosites being shifted towards higher or lower scores compared to the non-regulated (one-sided KS test).

### Kinases in AT_1a_R-HEK

NetworKIN supports predictions for 207 protein kinases. However, not all of the 207 kinases are expressed in AT_1a_R HEK293 cells used in the studies, while the algorithm does not support all expressed kinases. Therefore, we limited our analysis to the kinases in the overlap between the kinases supported by NetworKIN, the combined list of kinases found in the two experiments and an in-house AT_1a_R HEK293 proteome (Suppl. 2).

### Pathway analysis

In order to determine which pathways were affected downstream of the AT_1a_R, we analysed the 605 proteins with up-regulated phosphosites in Metacore (http://www.genego.com/metacore.php). The program analysed the list of proteins for pathway enrichment, using the MetaCore database as background.

## Supporting Information

Figure S1 **Plots of the p-value distribution.**
(PDF)

Figure S2 **A network map of the 5 most enriched pathways in the MetaCore software.** Angiotensin signalling map. A network map of the 5 most enriched pathways in MetaCore with an overlay of the angiotensin II pathway was produced in the MetaCoresoftware. This network, somewhat overwhelming, show the interactions between many of the proteins regulated in the angiotensin II phosphoproteome. And it reveals/indicates where some of the novel angiotensin signalling molecules fit in the angiotensin II pathway. A large portion of the proteins in the map have already been described in the angiotensin II signalling, thereby confirming the picture. The numbers represents proteins that were phosphorylated in response to SII Ang II (1) and Ang II (2).
(PDF)

Table S1 **Upregulated phosphosites in the two proteomics dataset.**
(XLSX)

Table S2 **NetworKIN predictions of the up regulated phosphosites.**
(XLSX)

Table S3 **The list of kinases in NetworKIN and AT_1a_R-HEK293.**
(XLSX)

Table S4 **The result of the Kolmogorov-Smirnof test of the up regulated phosphosites versus the non-regulated phosphosites.**
(XLSX)
