# Supplementary figures and images for: Predicting Kinase Activity in Angiotensin Receptor Phosphoproteomes Based on Sequence-Motifs and Interactions
Source: PLoS One. 2014 Apr 10;9(4):e94672. doi: 10.1371/journal.pone.0094672 (PMC3983226; doi:10.1371/journal.pone.0094672)

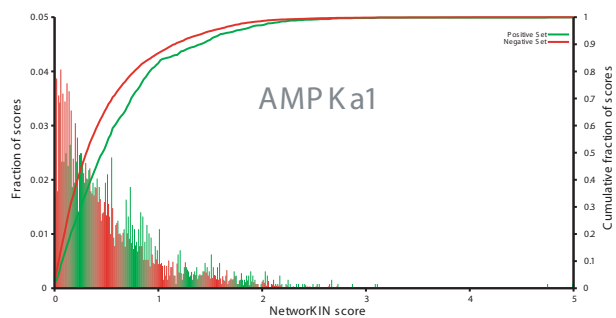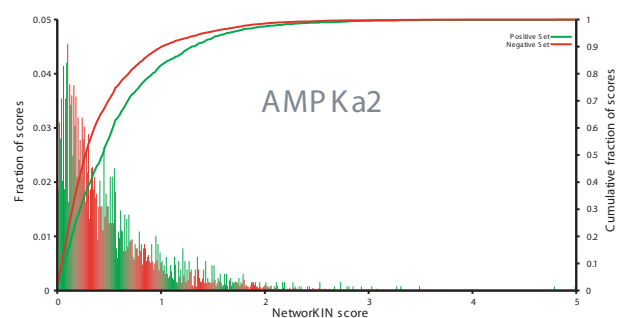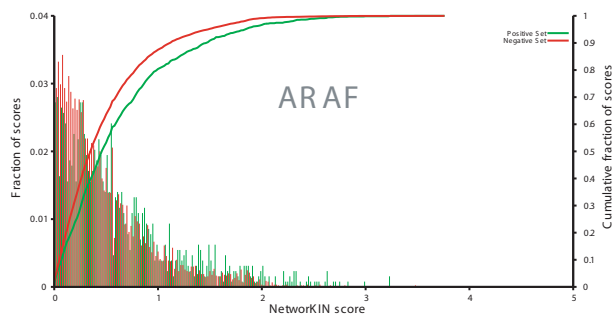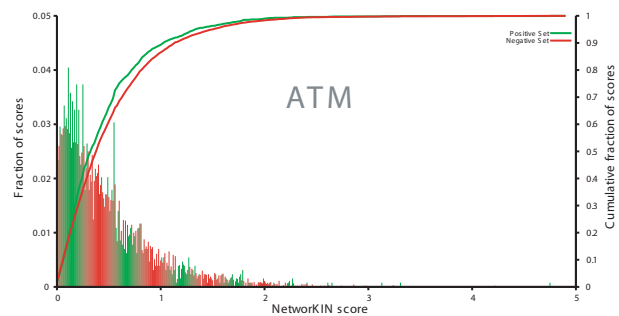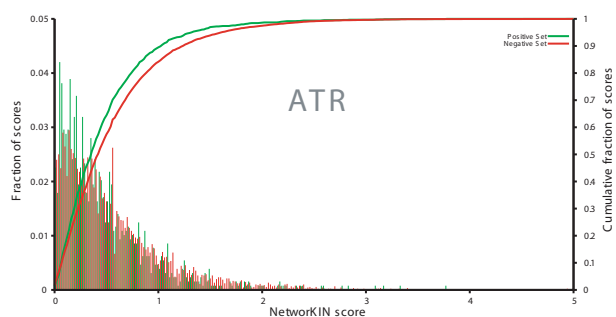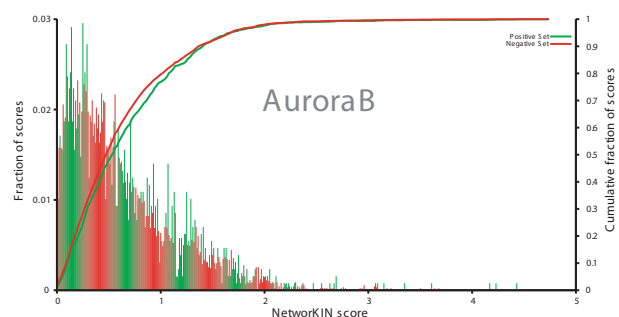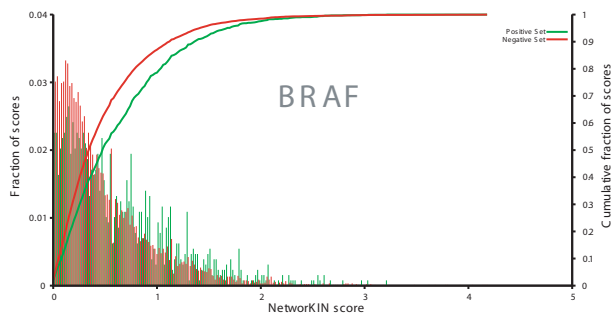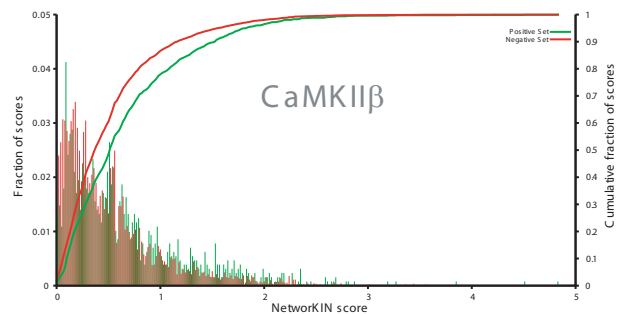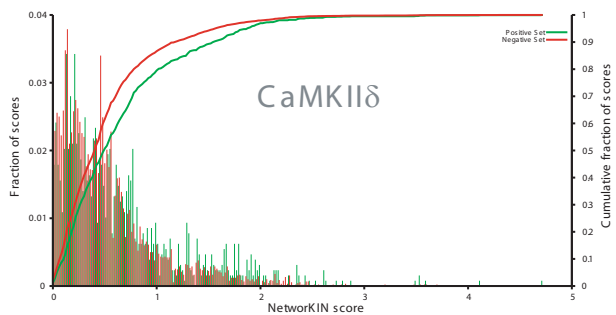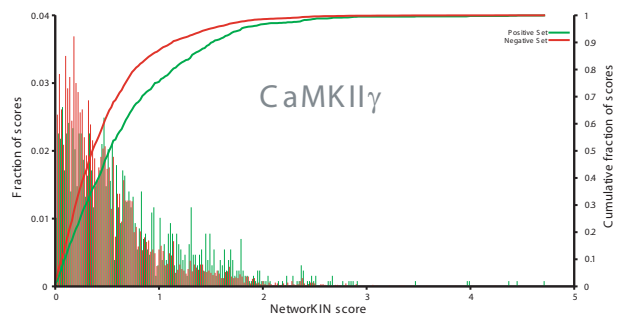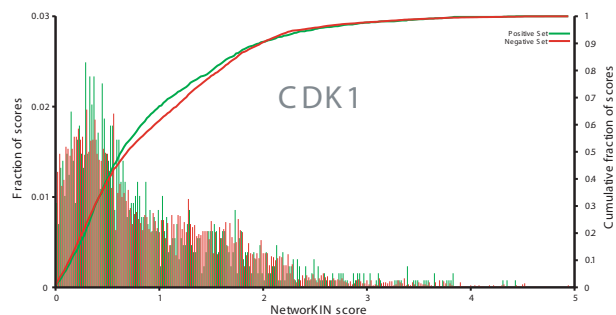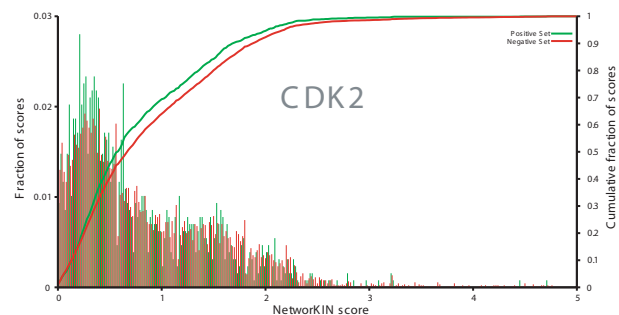

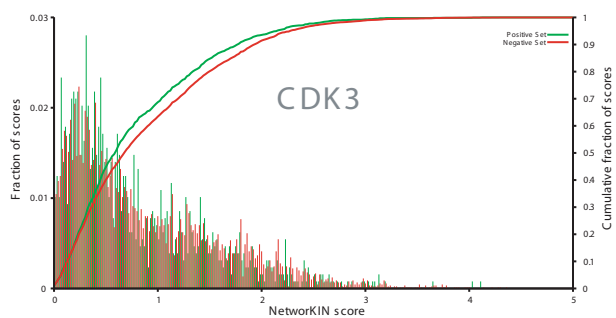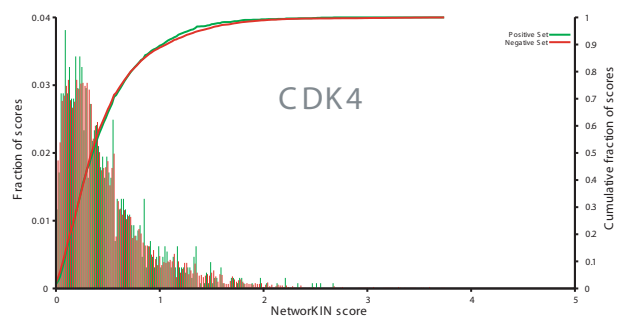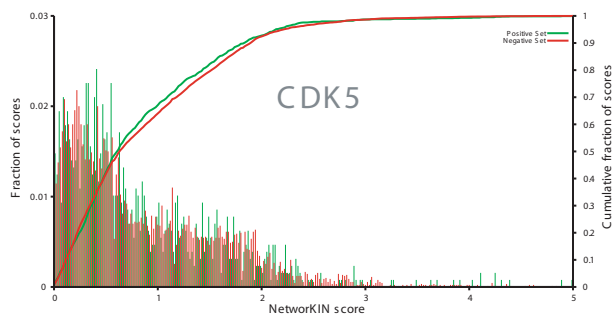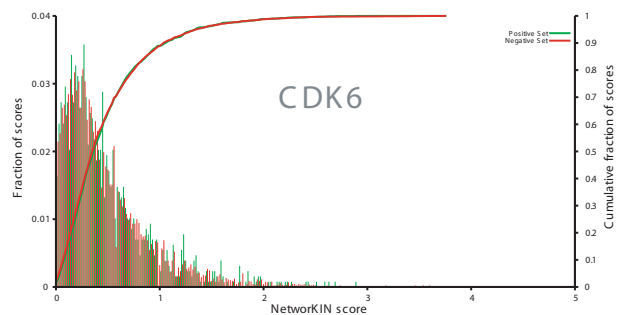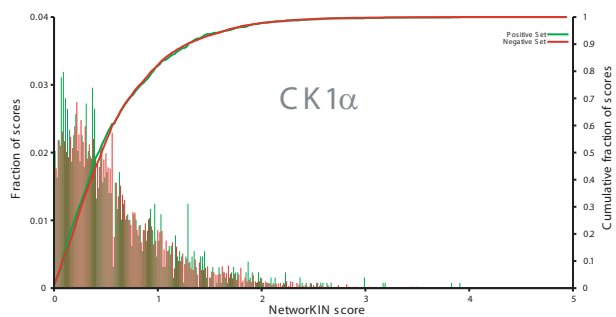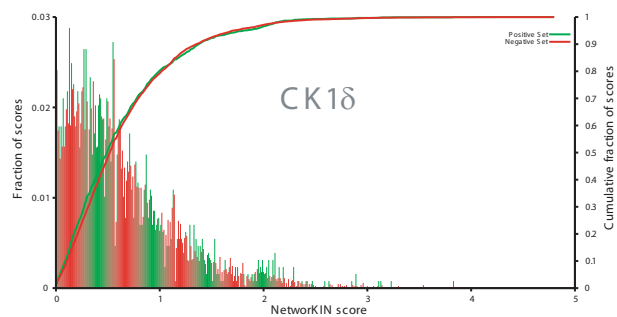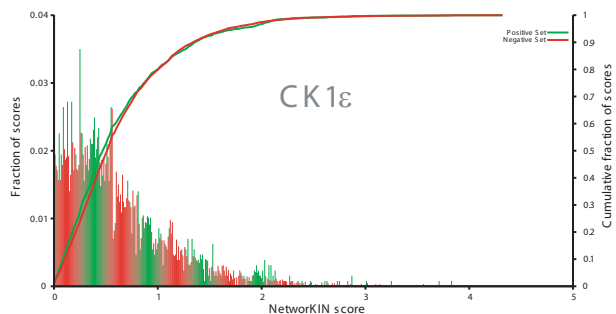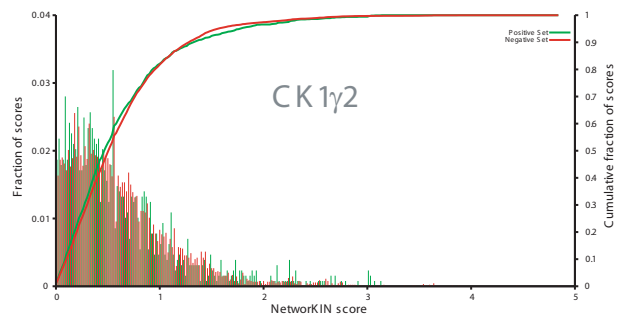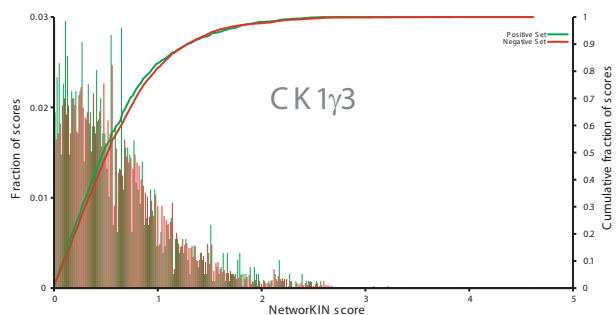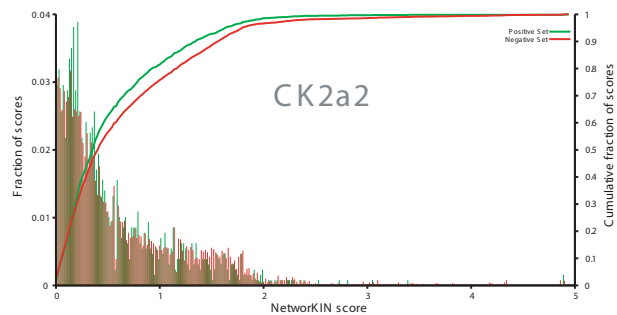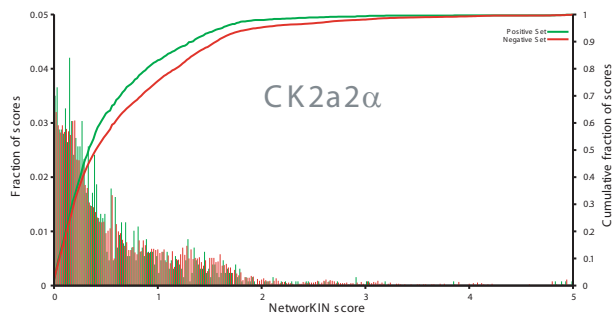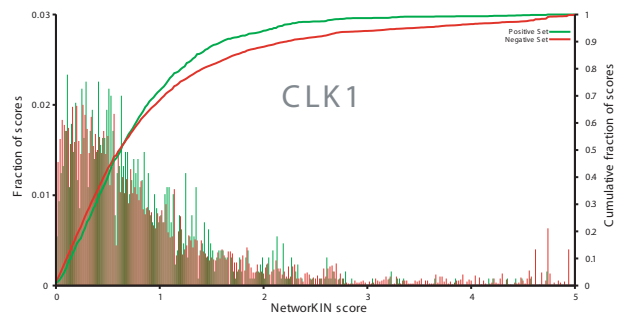

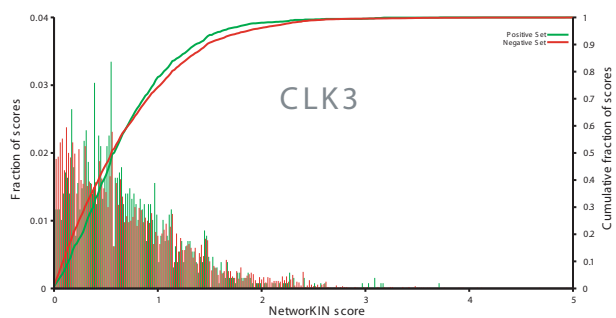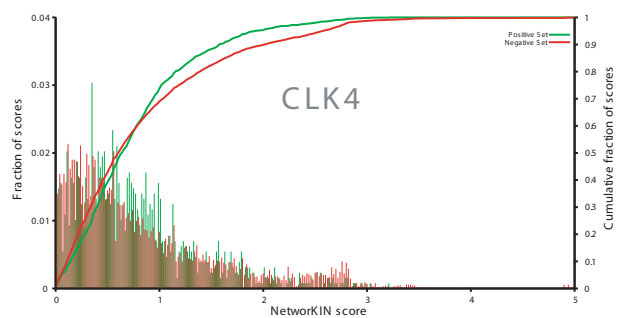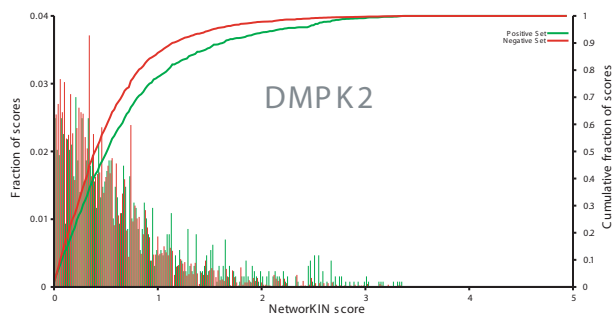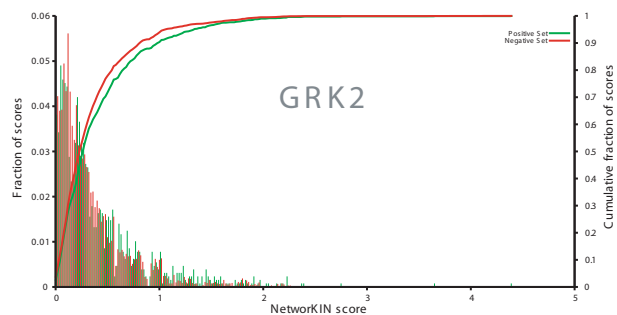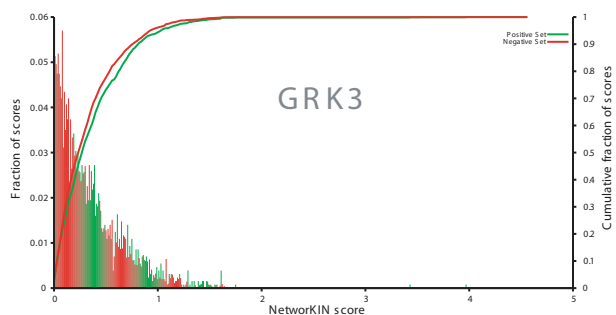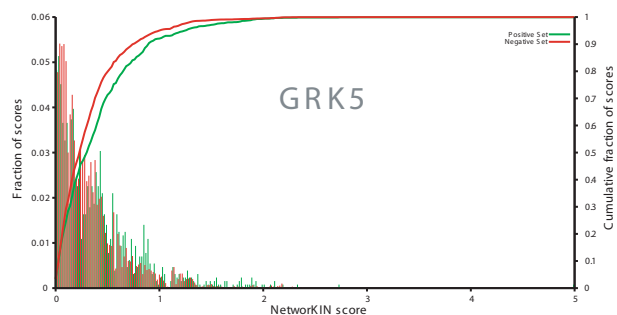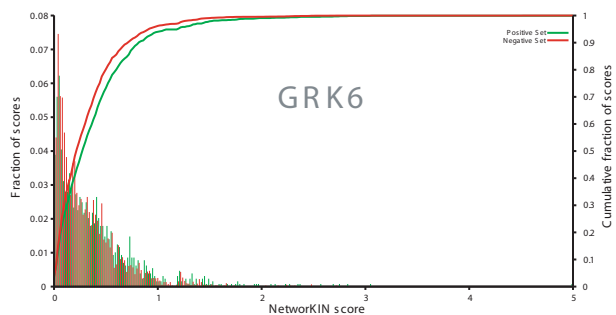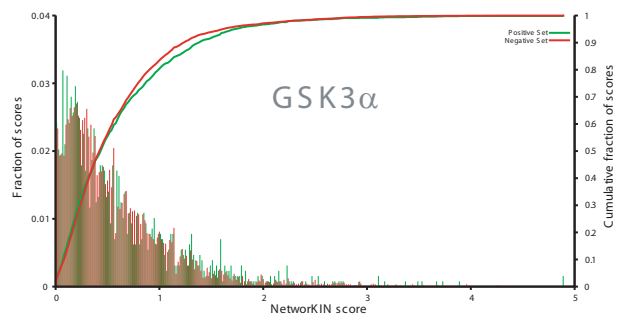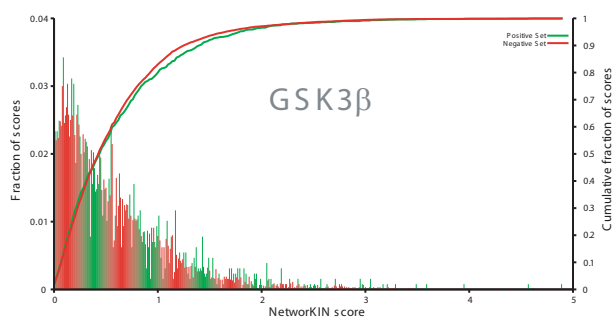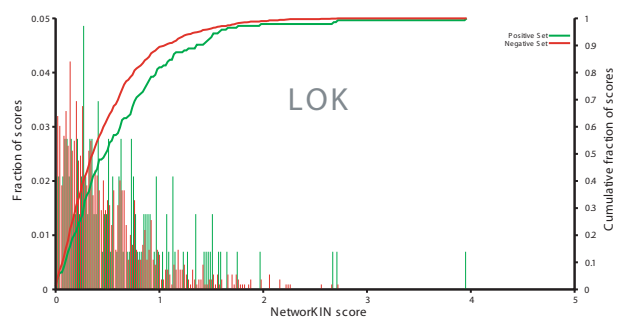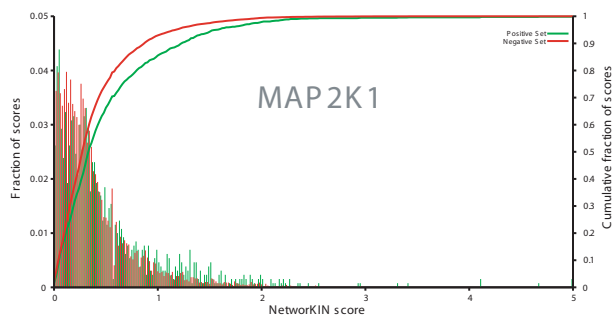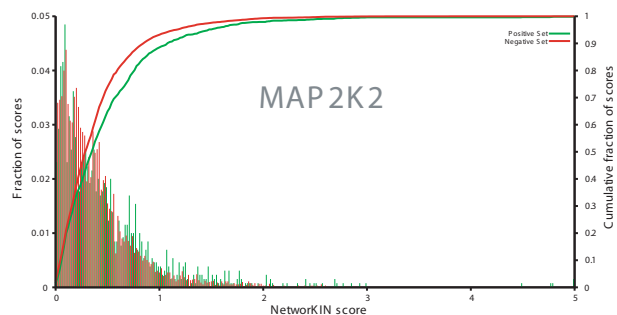

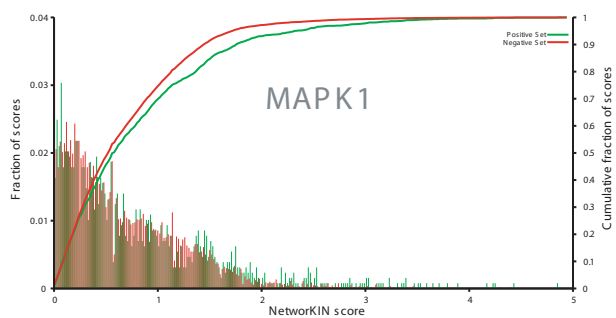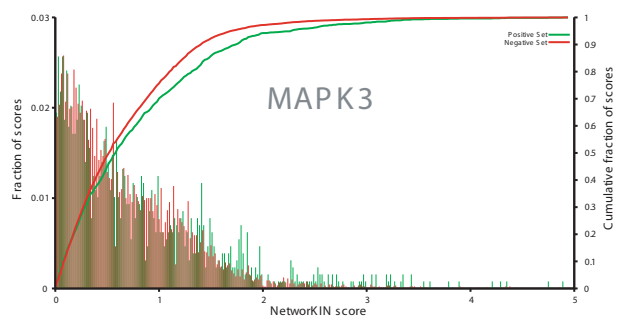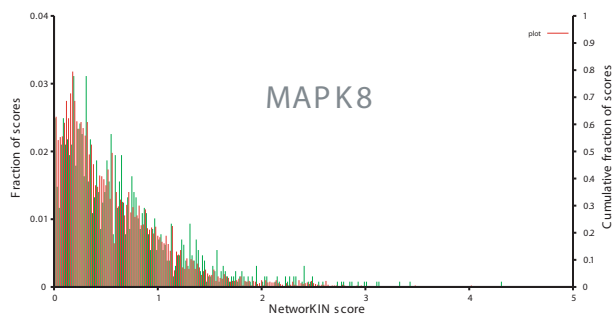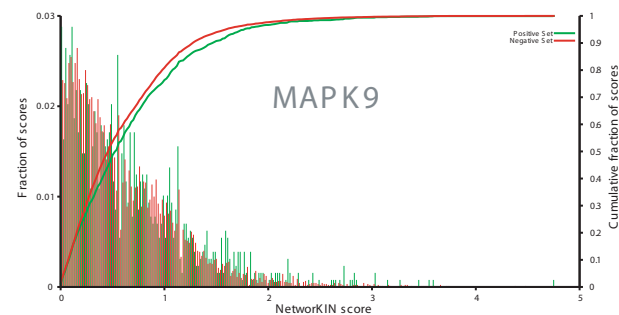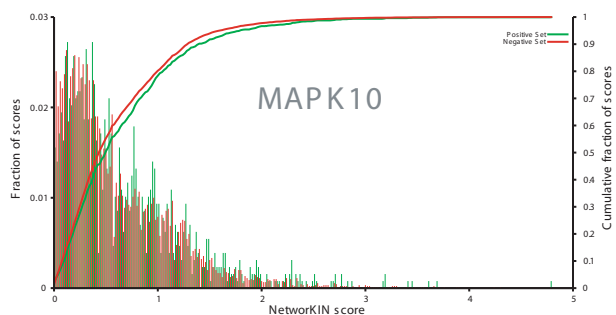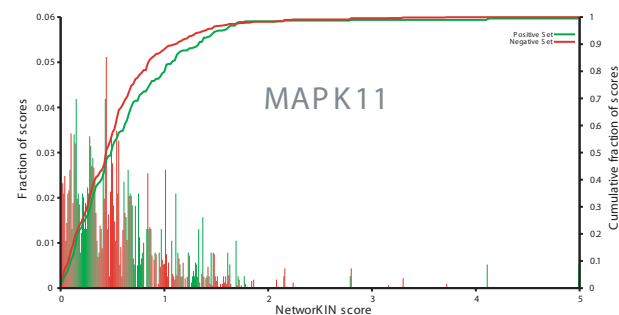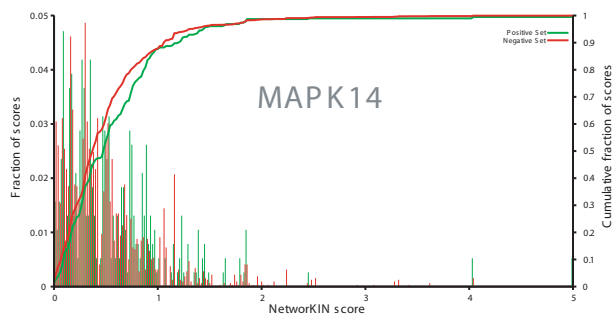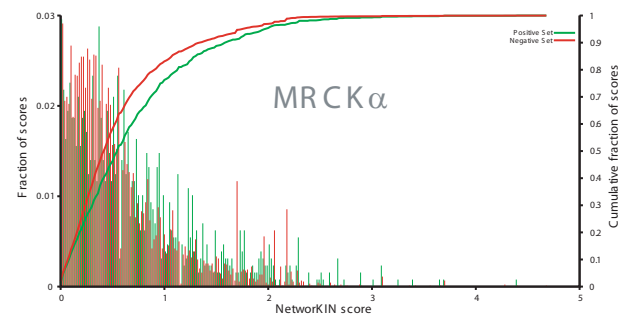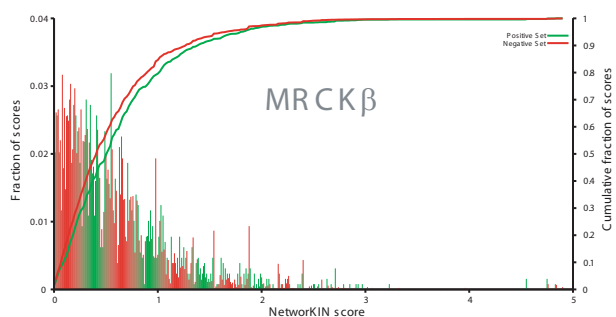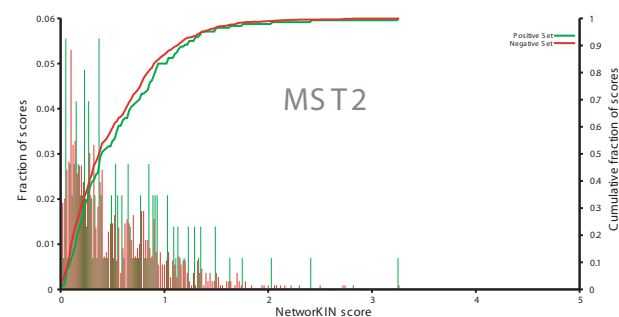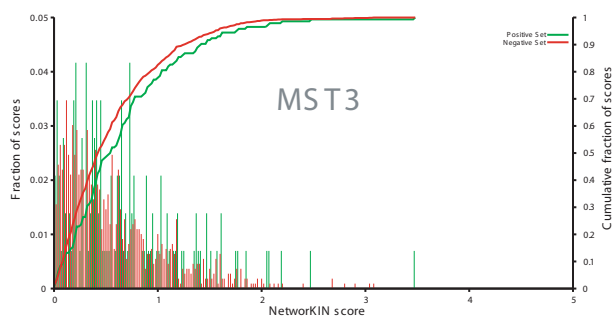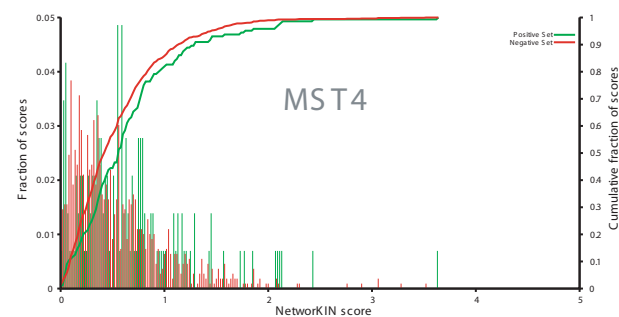

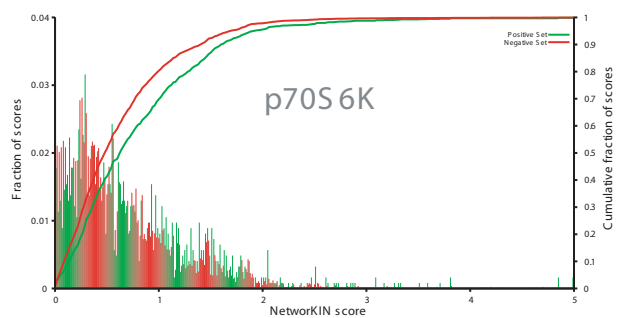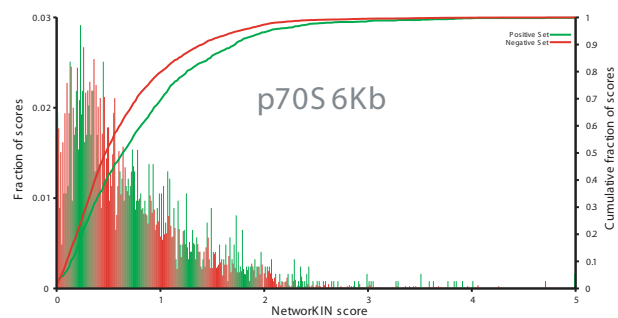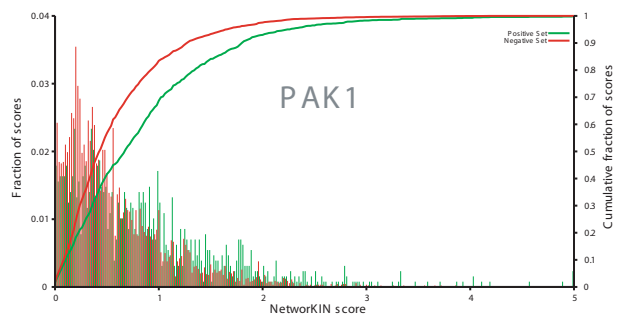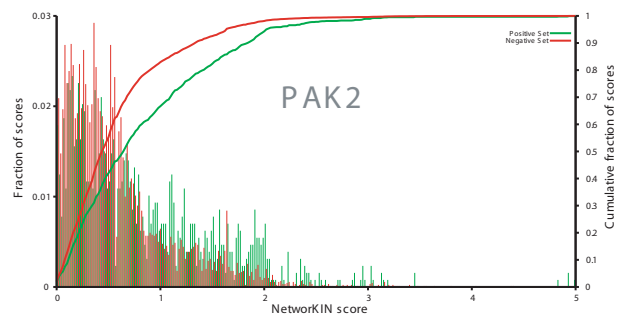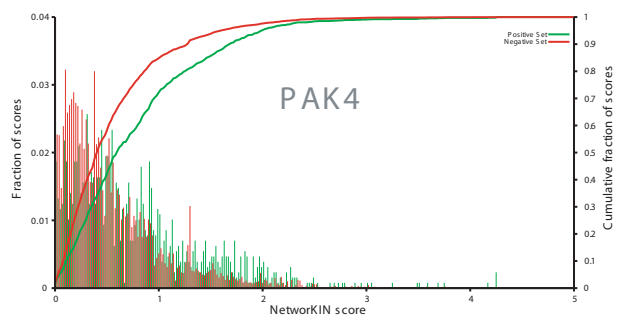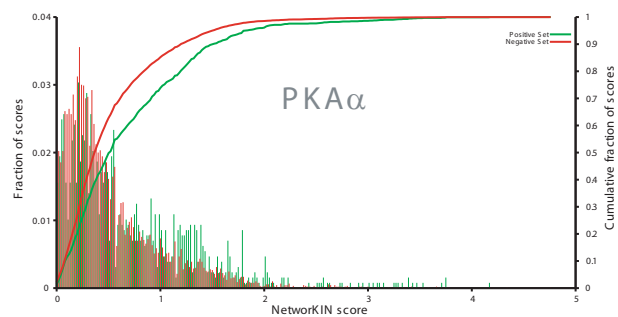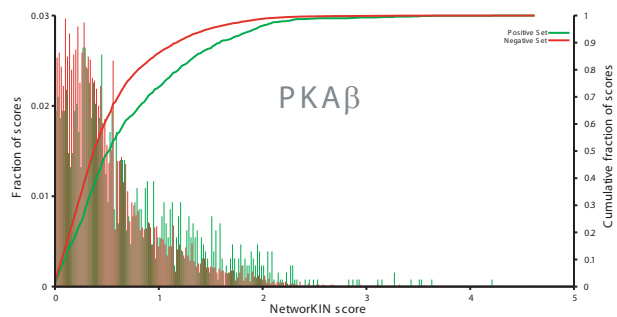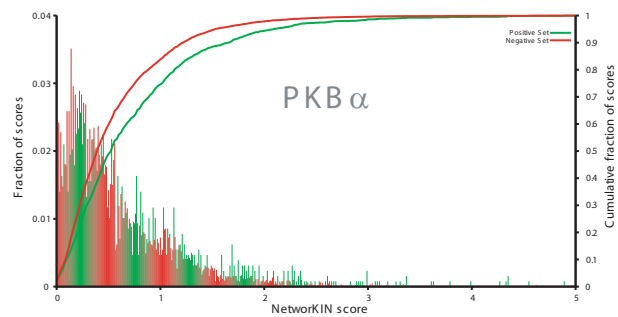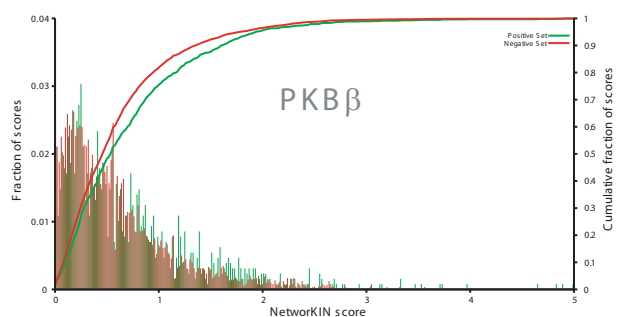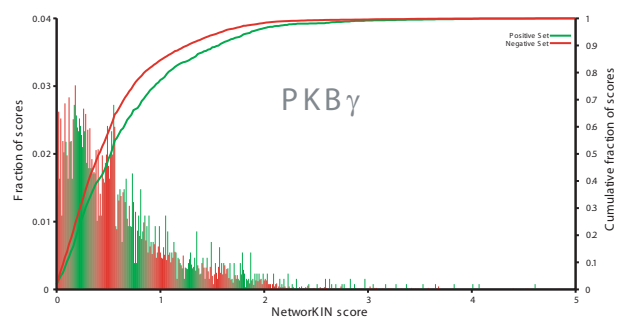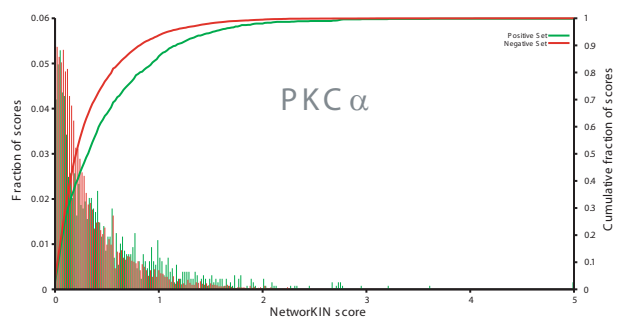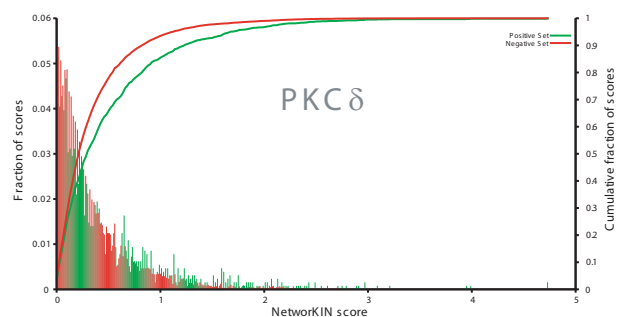

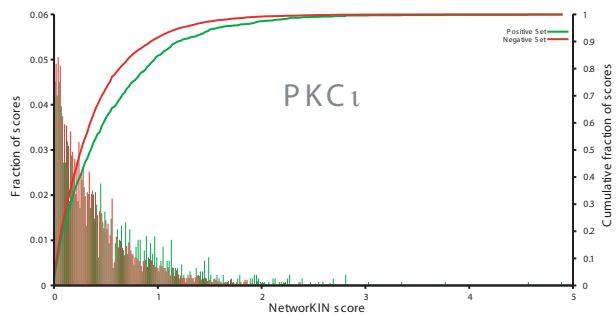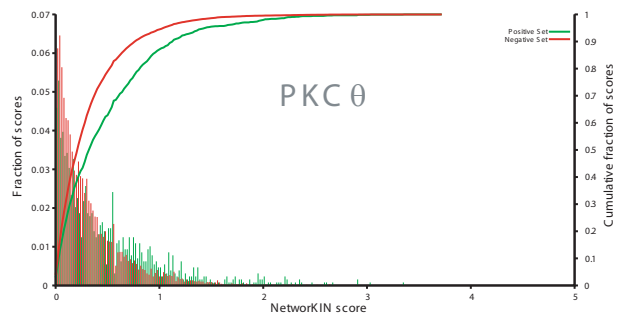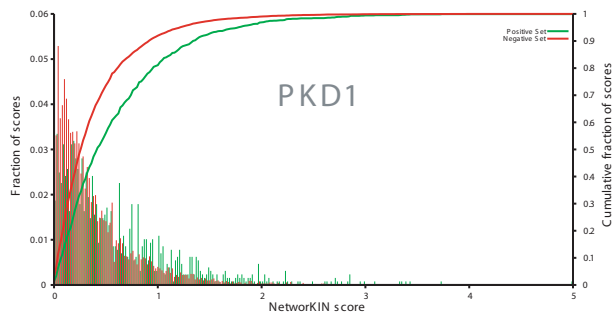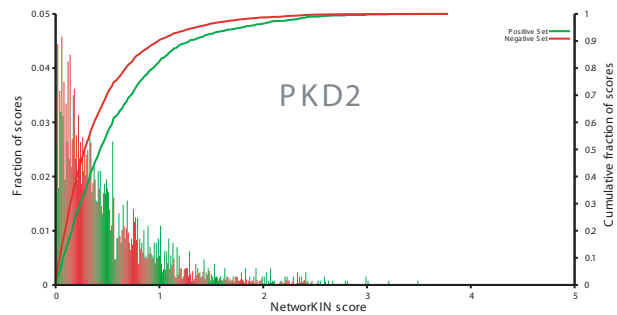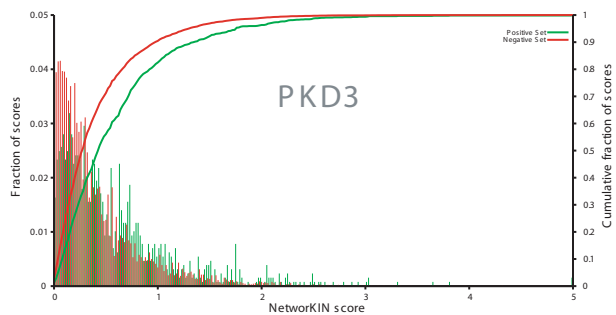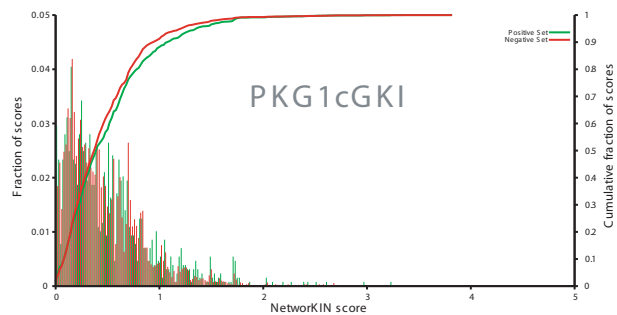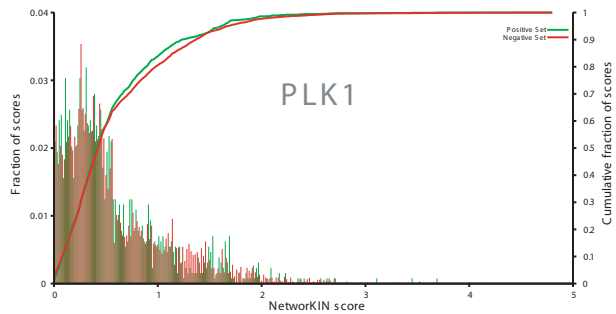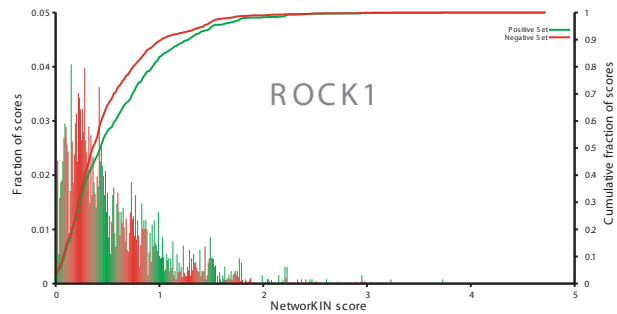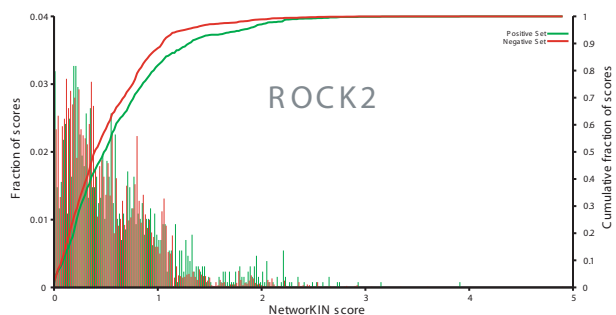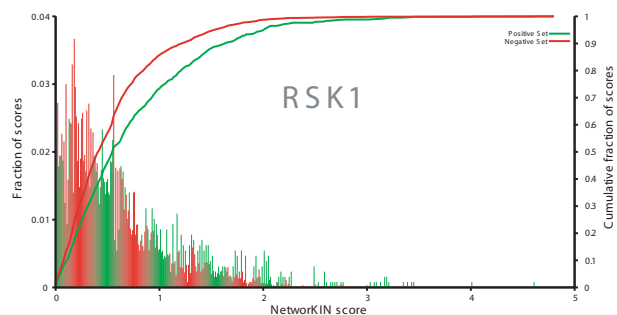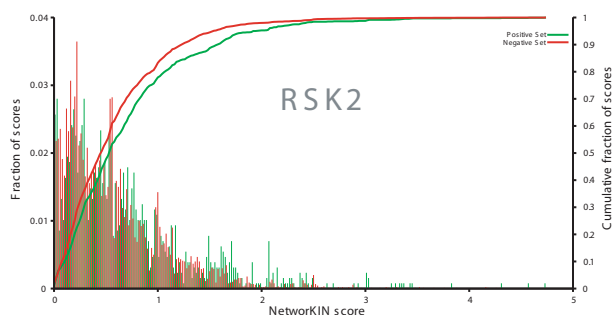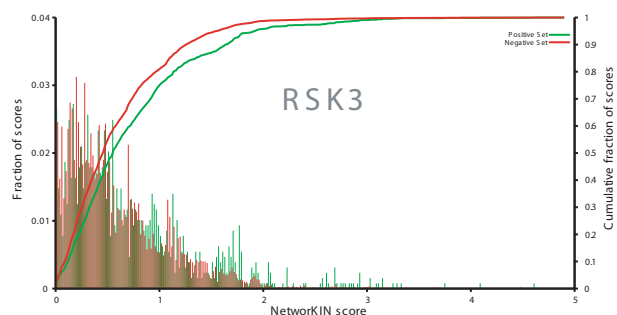

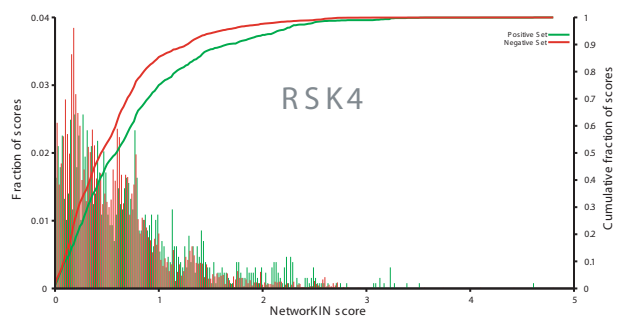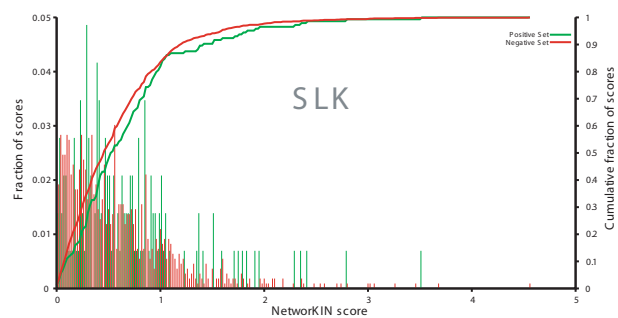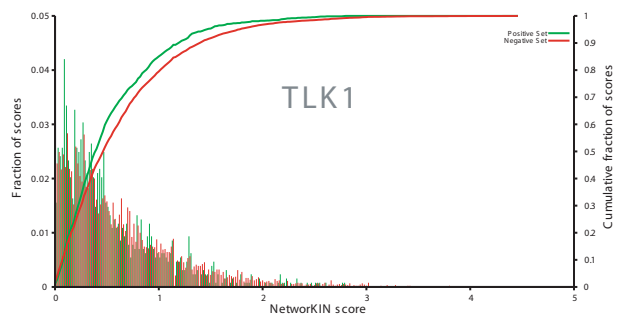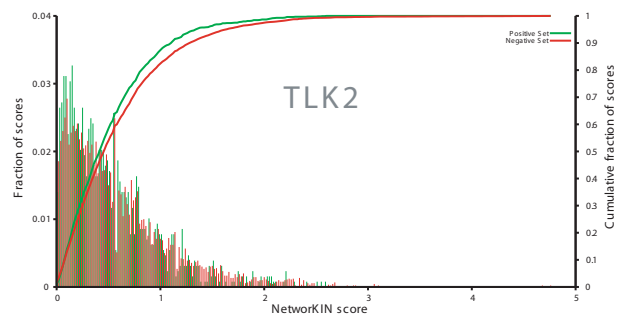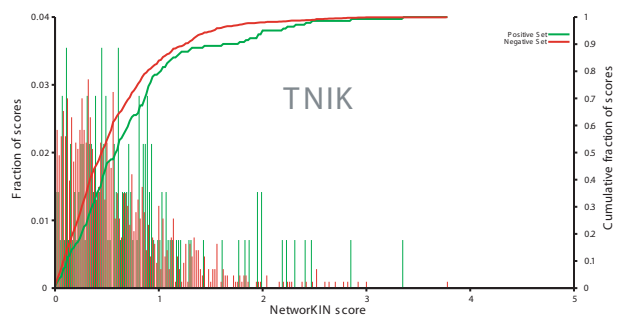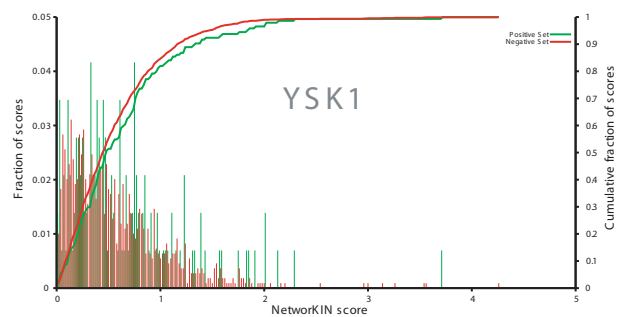

Supplement: Figure S1 — Plots of the p-value distribution. (PDF) [file pone.0094672.s001.pdf]

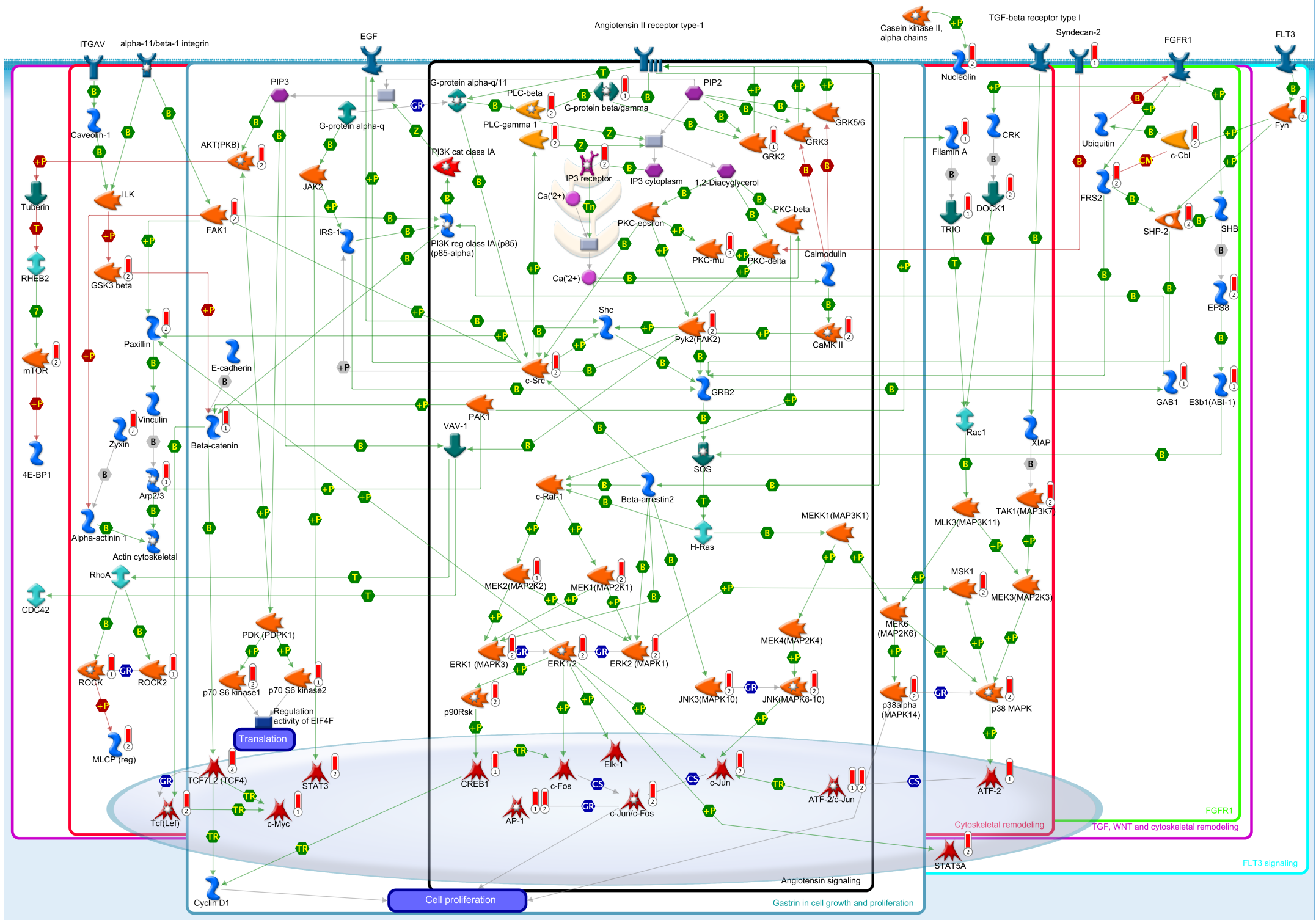

Supplement: Figure S2 — A network map of the 5 most enriched pathways in the MetaCore software. Angiotensin signalling map. A network map of the 5 most enriched pathways in MetaCore with an overlay of the angiotensin II pathway was produced in the MetaCoresoftware. This network, somewhat overwhelming, show the interactions between many of the proteins regulated in the angiotensin II phosphoproteome. And it reveals/indicates where some of the novel angiotensin signalling molecules fit in the angiotensin II pathway. A large portion of the proteins in the map have already been described in the angiotensin II signalling, thereby confirming the picture. The numbers represents proteins that were phosphorylated in response to SII Ang II (1) and Ang II (2). (PDF) [file pone.0094672.s002.pdf]
